# Disseminated *Fusarium keratoplasticum* Infection with Myocardial Involvement in an Adult Cord Blood Transplant Recipient

**DOI:** 10.1007/s11046-024-00900-y

**Published:** 2024-10-29

**Authors:** Masamichi Isobe, Seiko Kato, Masato Suzuki, Yasuhito Nannya, Satoshi Takahashi, Takaaki Konuma

**Affiliations:** 1 Department of Hematology/Oncology, The Institute of Medical Science, The University of Tokyo, 4-6-1, Shirokanedai, Minato-ku, Tokyo, 108-8639 Japan; 2 Department of Laboratory Medicine, Institute of Medical Sciences, The University of Tokyo, Tokyo, Japan; 3 Division of Clinical Precision Research Platform, The Institute of Medical Science, The University of Tokyo, Tokyo, Japan

**Keywords:** *Fusarium keratoplasticum*, Fusariosis, Fungemia, Myocarditis, Cord blood transplantation, Allogeneic hematopoietic cell transplantation

**Fig. 1:**
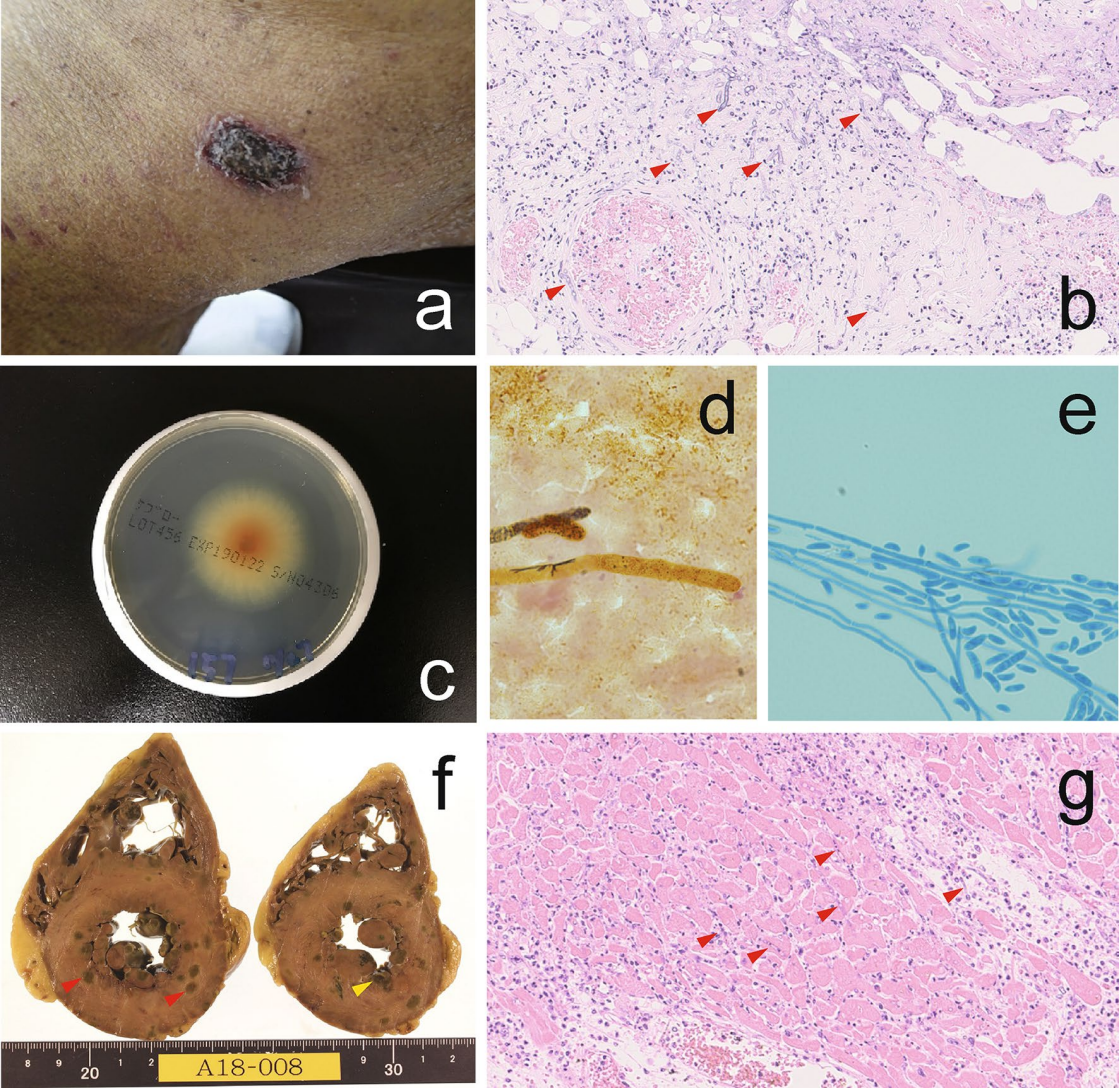
An erythematous nodule with a necrotic center on the neck (**a**). Hematoxylin and eosin stain of the skin necropsy. 400× magnification with accumulations of *F. keratoplasticum* hyphae in and around blood vessels with destruction and ulceration of blood vessels (**b**). Colony morphology after 10 days of incubation on Sabouraud dextrose agar (**c**). Microscopic structure of *F. keratoplasticum* hyphae. 1000× magnification with septate hyphae with branching at acute angles (**d**). 400× magnification with *F. keratoplasticum* stained with lactophenol cotton blue (**e**). The horizontal cut surface of the heart of the necropsy. Endocardium vegetation (yellow arrow) and multiple abscesses (red arrows) in the myocardial wall (**f**). Hematoxylin and eosin stain of the myocardium necropsy. 400 X magnification with *F. keratoplasticum* hyphae in the myocardium with surrounding lymphocyte and neutrophil infiltrate (**g**)

A 41-year-old man received a second unrelated single-unit cord blood transplantation (CBT) for acute myeloid leukemia that relapsed following the first CBT. Before the initiation of the conditioning regimen, liposomal amphotericin B (L-AMB) was started because of multiple lung nodules on a chest computed tomography (CT). Neutrophil engraftment was achieved on day 21, and a complete second donor chimerism was confirmed in bone marrow on day 35. Because of the grade 2 creatinine increment on day 12, cyclosporine was switched to prednisolone. On day 21, the patient developed watery diarrhea (> 1000 ml/day), suggesting that the patient developed grade III acute GVHD, and intravenous methylprednisolone was escalated up to 2 mg/kg/day on the same day. Despite administration of bone marrow-derived mesenchymal stem cells, high-dose methylprednisolone, and ruxolitinib under methylprednisolone treatment, the patients progressed to grade IV acute GVHD with liver stage 4 on day 53. Because of the grade 2 creatinine increment again, L-AMB was switched to micafungin (MCFG) on day 56. The levels of β-d-glucan in the serum slowly rose until they reached 27.27 pg/mL on day 67 (the upper limit of normal is 11 pg/mL), 75.52 pg/mL on day 74, and 483.6 pg/mL on day 77. Because the chest CT scan also revealed the progression of multiple lung nodules on day 68 along with an elevation of serum β-d-glucan levels, MCFG was switched to L-AMB. On day 78, the patient developed a low-grade fever and multiple erythematous nodules with a necrotic center on the neck and end of the limbs (Fig. 1a, b). Finally, the patient died of multiple organ failure on day 79 after the second CBT.

On day 79, the blood culture returned positive for Fusarium spp. growth 10 days after death (Fig. 1c, d, e). The internal transcribed spacer region gene sequence of the mold isolate in blood culture was completely identical (100%) to that of *Fusarium keratoplasticum* CBS490.63. The necropsy study revealed multiple abscesses in not only the lung and skin but also the thyroid gland, kidney, and myocardial wall (Fig. 1f, g).

*Fusarium* spp. are environmental filamentous fungi that cause disseminated fusariosis with fungemia in immunocompromised patients. Several studies showed that higher mortality from invasive fusariosis was associated with prolonged neutropenia and steroid therapy in hematological patients. In our case, prolonged steroid therapy for refractory acute GVHD following a second CBT could contribute to the development of disseminated fusariosis. *F. keratoplasticum* was identified in 2013, has melanin-producing ability, and is the second most common human *Fusarium* infection following *F. solani*. Melanin itself strengthens fungal host immunity and resistance to environmental changes, which could contribute to a dismal prognosis of disseminated fusariosis in our case.

Cardiac involvement in fusariosis is extremely rare. Peinado-Acevedo JS, et al. reported fatal endocarditis due to *F. keratoplasticum* in an adult with acute lymphoblastic leukemia under neutropenia following salvage chemotherapy. Along with our case, *F. keratoplasticum* is rarely reported as an etiological fungus of cardiac involvement.

